# Development stage of novel digital health interventions for postoperative monitoring: protocol of a systematic review

**DOI:** 10.1136/bmjsit-2021-000104

**Published:** 2022-03-04

**Authors:** Kenneth A McLean, Stephen R Knight, Thomas M Diehl, Syed Nabeel Zafar, Matt Bouamrane, Ewen M Harrison

**Affiliations:** 1 Centre for Medical Informatics, The University of Edinburgh Usher Institute of Population Health Sciences and Informatics, Edinburgh, UK; 2 Department of Surgery, University of Wisconsin-Madison School of Medicine and Public Health, Madison, Wisconsin, USA

**Keywords:** outcomes research, remote sensing technology, wireless technology, health services research, health care quality, access, and evaluation

## Abstract

**Introduction:**

The postoperative period represents a time where patients are at a high-risk of morbidity, which warrants effective surveillance. While digital health interventions (DHIs) for postoperative monitoring are promising, a coordinated, standardized and evidence-based approach regarding their implementation and evaluation is currently lacking. This study aimed to identify DHIs implemented and evaluated in postoperative care to highlight research gaps and assess the readiness for routine implementation.

**Methods:**

A systematic review will be conducted in accordance with Preferred Reporting Items for Systematic Reviews and Meta-Analyses guidelines to identify studies describing the implementation and evaluation of DHIs for postoperative monitoring published since 2000 (PROSPERO ID: CRD42021264289). This will encompass the Embase, Cumulative Index to Nursing and Allied Health Literature, Cochrane Library, Web of Science and ClinicalTrials.gov databases, and manual search of bibliographies for relevant studies and gray literature. Methodological reporting quality will be evaluated using the Idea, Development, Exploration, Assessment and Long-term Follow-up (IDEAL) reporting guideline relevant to the IDEAL stage of the study, and risk of bias will be assessed using the Grading of Recommendations, Assessment, Development and Evaluations (GRADE) framework. Data will be extracted according to the WHO framework for monitoring and evaluating DHIs, and a narrative synthesis will be performed.

**Discussion:**

This review will assess the readiness for implementation of DHIs for routine postoperative monitoring and will include studies describing best practice from service changes already being piloted out of necessity during the COVID-19 pandemic. This will identify interventions with sufficient evidence to progress to the next IDEAL stage, and promote standardized and comprehensive evaluation of future implementational studies.

## Introduction

As the capability and accessibility of mobile and wireless technologies continue to advance globally, digital health interventions (DHIs) to support delivery of healthcare have been widely regarded as essential for addressing current and future healthcare needs in both high-income countries and low-income and middle-income countries.^1^

The monitoring of patients after surgery represents a promising target for these interventions, representing a limited period of high morbidity which warrants increased attention.^2^ While Enhanced Recovery After Surgery programs have been effective at improving postoperative recovery (including reducing inpatient stay and complication rates), concerns remain on how to effectively identify and respond to potential complications in the community setting.^3^ The significant growth in internet access and digital literacy worldwide has enhanced the viability of DHIs for postoperative monitoring.^1 4^ However, there is a lack of coordinated, standardized and evidence-based approaches regarding development and evaluation of DHIs.^5^ These are common issues observed across many healthcare disciplines when developing complex health interventions.^6^ The Idea, Development, Exploration, Assessment and Long-term Follow-up (IDEAL) framework was established to address poor study design, inadequate reporting and research waste in clinical studies of surgical interventions at all stages of the innovation life cycle.^7^

Prior to the pandemic, there was widespread acknowledgement that the potential of DHIs had yet to be realized in healthcare.^1^ However, since the onset of the COVID-19 pandemic, the routine use of telemedicine has become a necessary and accepted practice across many healthcare settings.^8 9^ This has accelerated what may have otherwise taken decades of integrating telehealth into routine surgical practice and will likely form an important ongoing aspect of care even after restrictions lift.^8^ These complex interventions require not just evidence of effectiveness, efficacy and safety but also a clear understanding of how they might be best integrated into routine clinical practice.^6 10^ Therefore, this systematic review aimed to determine DHIs that have been developed and evaluated in postoperative care to date and so identify research gaps and/or readiness for routine implementation.

## Methods

The study protocol has been preregistered at the PROSPERO registry (CRD42021264289), and has been written according to the Preferred Reporting Items for Systematic Review and Meta-Analysis Protocols guidelines.^11^ Any deviations from this proposed methodology will be outlined and justified in the methodology of the resultant publication.

### Information sources and search strategy

A systematic literature search will be performed of key medical databases (Embase, Cumulative Index to Nursing and Allied Health Literature, Cochrane Library, Web of Science and Google Scholar) in accordance with Preferred Reporting Items for Systematic Reviews and Meta-Analyses guidelines. The search strategy was developed to identify all studies describing the implementation and evaluation of DHIs for postoperative monitoring (online supplemental file 1). This will be further supplemented through manual search of the bibliographies of relevant studies, gray literature and an international clinical trials register (ClinicalTrials.gov). The searches will be limited to publications in the English language due to practical restraints, and restricted to the year 2000 onwards to ensure relevance to current surgical practice and as previous systematic reviews in this sphere have not identified any relevant studies prior to 2000.^12–17^

10.1136/bmjsit-2021-000104.supp1 Supplementary data

### Eligibility criteria

The results of these searches, which will be uploaded onto the Covidence online systematic review tool,^18^ will facilitate efficient collaboration and will be screened for relevance against the eligibility criteria. Studies will be eligible for inclusion if they describe a primary study involving patients undergoing surgery (any procedure requiring a skin incision) and the use of a DHI for monitoring postoperative outcomes. DHIs are defined as interventions involving ‘the use of mobile and wireless technologies for health to improve health system efficiency and health outcomes’,^19^ and could be either the primary intervention or a component of an intervention. For example, these studies may range from those which use wearable technology to monitor postoperative physical activity or physiological signs, use smartphone cameras to allow remote review of surgical wounds or an online form to track patient quality of life postoperatively.

Studies excluded from the review will be (1) patients not undergoing surgery; (2) all reviews, commentaries/editorials, letters and conference abstracts; (3) all studies describing only the design requirements or protocol of a DHI; (4) interventions solely targeted at the anesthetic recovery period or DHIs which are already in routine use in healthcare (eg, continuous physiological monitoring in critical care); (5) all studies not meeting digital health criteria: (i) telehealth with stationary devices (eg, desktop videophone, desktop computer, videoconferencing equipment) unless the use of mobile devices or sensors are reported; (ii) telehealth which is web-based only; (iii) devices which are implanted; (iv) devices or interventions which are therapeutic; (v) devices for clinical diagnosis (eg, ECG) that did not report on a health outcome.

### Selection and data extraction

Following the removal of duplicate publications, initial screening of studies will be performed based on titles and abstracts by two independent investigators. In the event of disagreement between the two initial reviewers, studies will be evaluated by a third reviewer to establish a consensus. The same process will be adopted for full-paper screening.

Once the studies for inclusion have been determined, data will be extracted from each across four domains using a standardized data extraction instrument: (1) metadata and context of the study (article title, authors, publication year, journal, country and healthcare setting); (2) characteristics of the DHI (type of intervention; technology, infrastructure and platform used; duration of intervention; clinical outcome(s) assessed); (3) methodology of evaluation (study design, sampling methods, eligibility criteria and patient population (eg, age, sex, operation type and surgical specialty)); and (4) results of the evaluation in regard to the WHO evaluation framework for monitoring and evaluating DHIs^20^ (eg, functionality and feasibility; usability (the quality of the interaction between the user and the technology); process improvement (how the technology improves service delivery at patient, provider and health-system levels); clinical efficacy; and other measures of evaluation reported). Data extraction will be performed independently by each reviewer (with random selection cross-checked by a third reviewer) and will be stored on a secure Research Electronic Data Capture server.^21^

Furthermore, studies that do not already report their stage under the IDEAL framework for surgical innovation^7^ will be retrospectively assigned a stage based on the methodology reported (table 1).

**Table 1:** Criteria used to assess study design according to the IDEAL framework^7^

IDEAL stage	Identifying study characteristics
1 (Idea)	Case report.
2 a (Development)	Single-center/single intervention; case series/ prospective cohort.
2b (Exploration)	Prospective multicenter exploration cohort study or pilot/feasibility multicenter RCTs.
3 (Assessment)	Full-scale RCT which involves a comparison to routine clinical practice.
4 (Long-term)	Long-term evaluation of the implementation within routine clinical practice using registries or databases.

IDEAL, Idea, Development, Exploration, Assessment and Long-term fFollow-up; RCT, randomized controlled trial.

### Quality assessment

The quality of reporting for included studies will be performed using the IDEAL collaboration guideline relevant for the IDEAL classification assigned to the study.^22^ Each component will be categorized as either being fully addressed, partially addressed, not addressed or not applicable. The Grading of Recommendations, Assessment, Development and Evaluations (GRADE) approach will be used to assess for bias across all studies, if a sufficient number are included.

### Data synthesis

Narrative (descriptive) synthesis of results will be performed, with the data extracted to be summarized using the appropriate descriptive statistics: frequencies and percentages for dichotomous variables, and mean (SD) or median (IQR) for continuous variables. Furthermore, the DHIs will be classified into subgroups based on the outcome under investigation: (1) general postoperative recovery and/or rehabilitation, (2) surgical wound care and (3) other specified postoperative complications. The results of the evaluation of each intervention will be explored within these subgroups. Further subgroup analysis by country income level (high income vs low income and middle income) are also planned, if a sufficient number are included in each group.

## Discussion

Digital postoperative monitoring poses an immense opportunity to understand and improve postoperative community care and reduce the burden of disease for both patients and healthcare services. However, these benefits have yet to be realized and still face major challenges in how these can be effectively and safely implemented in routine clinical practice. In addition to demonstrating the clinical efficacy and safety of these interventions, there must be consideration of how to ensure appropriate stewardship of data collected,^23^ how to ensure accessibility and acceptability across all stakeholders,^24^ and how health systems may be required to be restructured to deliver and benefit from these interventions.^25^

To our knowledge, this is the first systematic review to examine the implementation and evaluation of all DHIs for postoperative monitoring, and to apply the IDEAL reporting guidelines to assess the literature.^22^ This contrasts to similar previous reviews which have typically focused on a specific type of intervention (whether smartphone^12–15^ or telemetry^17^) or only the clinical efficacy rather than the implementation of these interventions.^12–16^ Furthermore, this review represents a novel application of the IDEAL framework to examine DHIs for postoperative monitoring. This is a rapidly evolving field, with progress of implementation within routine practice accelerated by the COVID-19 pandemic.^8 9^ Therefore, this review will provide a timely assessment of the current evidence base and readiness for implementation for DHIs, including studies describing best practice from service changes already being piloted out of necessity during the ongoing COVID-19 pandemic.^26^ This review will support ongoing efforts to minimize research wastage by identifying interventions with sufficient evidence to progress to the next IDEAL stage, and to promote standardized and comprehensive evaluation of future implementational studies.

There are also several key challenges anticipated. First, while there may be common outcomes for postoperative monitoring of DHIs, there is expected to be significant heterogeneity in the literature in terms of the interventions developed, and the quality of studies and their reporting in publications. Therefore, this may limit definitive conclusions if results present a high risk of bias or are not applicable outside their local context, limiting the wider clinical relevance. Second, there is currently no ‘gold-standard’ framework to evaluate the implementation of DHIs,^27^ likely leading to heterogenous measures reported. In order to facilitate meaningful comparison between studies, the WHO evaluation framework for monitoring and evaluating DHIs^20^ has been used as the basis for this review due to its applicability across different income settings and healthcare contexts. This provides several recommended domains and indicators for which to evaluate DHIs against: functionality and feasibility of the technology itself within the healthcare context; usability by patients and healthcare staff; and evidence of improvement to service delivery for either patients, healthcare staff or systems (whether with regard to the healthcare outcomes, or the quality, efficiency, use or overall cost of healthcare). This will allow identification of areas currently overlooked in the literature with regard to the evaluation of digital postoperative monitoring. Finally, it must be acknowledged that published evidence may not represent the breadth of implementation of DHIs performed, leading to publication bias favoring successful interventions or those performed at academic and/or highly resourced centers.
