# Supplementary material for: Development stage of novel digital health interventions for postoperative monitoring: protocol of a systematic review
Source: BMJ Surg Interv Health Technol. 2022 Mar 4;4(1):e000104. doi: 10.1136/bmjsit-2021-000104 (PMC8900039; doi:10.1136/bmjsit-2021-000104)
Supplement: Supplementary data [file bmjsit-2021-000104supp001.pdf]

Supplementary File: Search Strategy

EMBASE / CINAHL / WHO Global Index Medicus

1. exp cellular phone/
2. exp microcomputers/
3. (smart phone$ or smartphone$ or iphone$ or android or blackberry$).tw.
4. ((handheld or portable or mobile) adj2 (phone$ or device$)).tw.
5. (personal digital assistant$ or pda).tw.
6. (ipad$ or tablet$).tw.
7. text messag$.tw.
8. sms.tw.
9. (e-health or telemedicine or digital health or wearable* or mobile health or mHealth or digital health or eHealth or LORAWaN).ti,ab.
10. or/1-9
11. (surgery or surg*).ti,ab.
12. (oper$ or post$).ti,ab.
13. 11 and 12
14. 10 and 13
15. limit 14 to (human and english language and yr="2000 - Current")
16. limit 15 to (article or article in press or "review")

The Cochrane Library

1. MeSH descriptor: [Cellular Phone] explode all trees
2. MeSH descriptor: [Microcomputers] explode all trees
3. ("smart phone*" or smartphone* or iphone* or android or blackberry*):ti,ab
4. ((handheld or portable or mobile) near/2 (phone* or device*)):ti,ab
5. ("personal digital assistant*" or pda):ti,ab
6. (ipad* or tablet*):ti,ab
7. "text messag*":ti,ab
8. sms:ti,ab
9. (e-health or telemedicine or digital health or wearable* or “mobile health” or mHealth or “digital health” or eHealth or LORAWaN):ti,ab
10. #1 or #2 or #3 or #4 or #5 or #6 or #7 or #8 or #9
11. surgery or surg*:ti,ab
12. (oper* or post* or outcome* or peri$ or “peri-operative period”):ti,ab
13. #11 and #12
14. #10 and #13

Clinicaltrials.gov

- mHealth AND surgery AND completed studies
- mobile AND surgery AND completed studies
- phone AND surgery AND completed studies
- pda AND surgery AND completed studies
- text AND surgery AND completed studies
- txt AND surgery AND completed studies
- ipad AND surgery AND completed studies

WHO International Clinical Trials Registry Platform / Web of Science

mHealth or mobile or phone or pda or text or txt or ipad and surgery in Intervention
